# Outcomes of non-operatively managed Vancouver Type B1 periprosthetic femur fractures: a multi-center retrospective cohort study

**DOI:** 10.1186/s12891-025-08535-w

**Published:** 2025-04-08

**Authors:** Mitchell Crebert, Michael Le, Geoff Murphy, Annamaria Frangos Young, Robert Molnar, Daniel Franks, Michael Symes, Maurice Guzman

**Affiliations:** 1 Department of Orthopaedics, Royal Prince Alfred Hospital, Camperdown, Australia; 2 Faculty of Medicine, The University of Sydney, Sydney, Australia; 3 Faculty of Medicine, The University of New South Wales, St George and Sutherland Clinical Schools, Kogarah, Australia; 4 Department of Orthopaedics, Royal North Shore Hospital, Camperdown, Australia; 5 Department of Orthopaedics, St George Hospital, Kogarah, Australia; 6 Sydney Orthopaedic Trauma and Reconstructive Surgery, Kogarah, Austria; 7 Campbeltown Hospital, Campbelltown, Austria

**Keywords:** Periprosthetic fracture, Hip arthroplasty, Mortality, Nonoperative, Vancouver B

## Abstract

**Background:**

This retrospective case series evaluated mortality outcomes in patients with Vancouver B1 periprosthetic fractures (PPFs) managed non-operatively using a matched cohort approach. We hypothesize that mortality rates will not significantly differ between operative and non-operative management of Vancouver B1 PPFs, as treatment decisions are likely driven by fracture complexity and patient comorbidities rather than a direct survival benefit of surgical intervention.

**Methods:**

Thirty patients with Vancouver B1 PPFs managed non-operatively between 2011 and 2017 across five major Australian trauma centers were identified. Patients were propensity-matched to 60 operatively managed patients, matched by age, ASA score, length of stay, follow-up duration, and fracture sub-type (B1). Mortality rates at 30 days, 1 and 5 years were compared between the non-operative and operatively managed groups. For the non-operative group alone, the impact of weight-bearing status on mortality was assessed.

**Results:**

There was no significant difference in mortality rates between the non-operative and operative cohorts at 30-day (3.3%; 1.7%; *P =* 1.00), 1 year (20.0%; 3.3%; *P =* 0.09) and 5 years (33.3%; 30.0%; *P =* 0.78). For the non-operative group alone, there was no significant difference in mortality rates between WBAT and non-WBAT groups at 30 days (7.7%; 0.0%; *P* = 0.400), 1 year (15.4%; 17.6%; *P* = 0.839) and 5 years (30.8%; 35.3%; *P* = 0.781),

**Conclusion:**

Comparable 5-year mortality rates were identified between non-operatively and operatively managed Vancouver Type B1 periprosthetic femoral fractures. Despite differences in age and comorbidities, non-operative management may be a viable option for selected patients, underscoring the need for further research to refine treatment guidelines.

**Clinical trial number:**

Not applicable.

## Introduction

Between 2012 and 2022 the estimated incidence of total hip arthroplasty (THA) in the US increased by 66% [1]. An increase in THA has been accompanied by a proportional increase in post-operative complications, including periprosthetic femoral fractures (PPF) [2]. These fractures occur adjacent to the prosthetic implant and are classified based on their location relative to the femoral component [3]. The Vancouver classification system is widely used to categorize these fractures and assists in the selection of appropriate management modalities for patients [4]. Vancouver Type A fractures involve the proximal metaphysis, without extension into the diaphysis of the femur [4]. Type B fractures occur around the femoral stem, whereas Vancouver Type C fractures occur well distal to the femoral stem [3]. Type B fractures are then categorized on the basis of the stability of the femoral stem, B1 (stable stem), B2 (loose stem) and B3 (loose stem with poor bone stock) [4]. With an incidence between 1 and 5% following THA, periprosthetic femur fractures pose a significant challenge in orthopedic surgery, particularly in cases classified as Vancouver Type B fractures [3]. These fractures occur around the femoral component of a THA and can have a profound impact on patient mobility, functional capacity and quality of life [5, 6]. 

While surgical intervention has traditionally been the standard approach, there is growing interest in exploring non-operative management strategies for certain cases, particularly patients who are medically unfit for surgery due to advanced age, have multiple comorbidities or have minimally displaced fractures [7, 8]. 

There is a paucity of literature on the nonoperative management of Vancouver B PPFs. Specifically, studies that focus on nonoperative management for Vancouver B1 fractures are limited to 1-year mortality data [8–10]. While providing insights into the sub-acute risks of managing patients nonoperatively, clinical uncertainty remains around the success of this management modality on long-term mortality outcomes and how they compare to surgically managed patients [8–10]. Furthermore, nonsurgical management is relatively uncommon, and significant variability in patient and fracture characteristics, combined with small sample sizes, makes it difficult to identify appropriate candidates for this approach [8–10]. 

In this case series, we investigated the outcomes of 30 patients with nonoperatively managed Vancouver Type B1 periprosthetic femoral fractures (PPFs). Specifically, we compared mortality rates at 30 days, and at 1 and 5 years to those of matched surgically managed patients. Our secondary aim was to assess the effects of weight-bearing status on mortality rates, contributing to the understanding of optimal management strategies for this challenging orthopedic condition. Finally, we aimed to identify patient and implant characteristics associated with successful outcomes in nonoperatively managed Vancouver B1 PPFs. We hypothesize that there will be no significant difference in mortality rates between operatively and non-operatively managed Vancouver B1 periprosthetic femoral fractures, as treatment decisions are likely influenced by patient comorbidities, rather than a direct survival benefit of surgical interventions.

## Methods

This study was conducted in adherence to the principles outlined in the Declaration of Helsinki [11]. Ethical approval was obtained from the Sydney Local Health District Human Research Ethics Committee (Approval Number: 2023/ETH01168).

A retrospective review of the databases of five major Australian trauma centers, namely Liverpool Hospital, St George Hospital, Sutherland Hospital, Royal North Shore Hospital, and Royal Prince Alfred Hospital to identify all Vancouver B PPFs between 2011 and 2017. All demographic, operative, and imaging data were systematically compiled in the Sydney Local Health District Research Data Capture (REDCap) system. Data entries were fully de-identified and secured with encrypted, password-protected access to ensure confidentiality and data integrity. A total of 239 cases were identified across both surgical and nonsurgical managed cases.

Fracture patterns were classified using the Vancouver system, based on either operative reports at the time of surgery or independent pre-operative radiographic reviews by two experienced orthopedic trauma surgeons. A standardized assessment template was employed to evaluate fracture characteristics, including subsidence, radiolucency around the prosthesis, stem position, and fracture morphology. Additionally, pre-operative clinical notes were reviewed for documentation of pain and relevant physical examination findings. Any disagreements on Vancouver classifications were resolved by a senior orthopedic consultant and a final decision was made. All patients had a minimum of 5 years follow-up. Patients with non-surgically managed periprosthetic femur fractures were identified through queries of our trauma database and billing records. Patients with isolated fractures of the greater or lesser trochanters (Vancouver A) or fractures well distal to the femoral stem (Vancouver C) were excluded.

A standardized and piloted template was used to extract patient demographic details including age, gender, co-morbidities, residence on admission (independent residence or nursing home), initial indication for the implant and American Society of Anesthesiologists (ASA). Additionally, surgical interventions, and post-operative data such as weight bearing status, fracture union, length of stay and residence on discharge were meticulously collected through a review of medical records and radiographs. Based on this the Charlson Comorbidity Index (CCI) was retrospectively calculated for each patient. Mortality data were retrieved from hospital records linked to individuals’ Medicare numbers.

Non-surgically managed patients were propensity matched to surgically managed Vancouver B1 fracture patients based on age, length of stay (LOS), ASA score, and follow-up duration. Mortality rates at 30 days, 1 year, and 5 years were then compared between the two cohorts.

The primary outcome measure focused on mortality outcomes at 30-days, 1-year and 5-year follow-up, for patients managed non-operatively for Vancouver Type B1 fractures, which were recorded through thorough examination of medical records. The secondary outcome measure assessed the impact of weightbearing status on mortality.

### Statistical analysis

All statistical analyses were performed on an intention-to-treat basis. Continuous parametric data was analyzed using unpaired Student’s t tests and Chi-Squared tests for categorical data. Significance was set at *p* > 0.05 for all tests. Statistical analysis was performed using SPSS (v26).

A post-hoc power analysis was conducted using SPSS (v26) to assess the adequacy of the sample size in detecting differences in 30 day, 1 and 5-year mortality between the operative and non-operative groups.

## Results

A total of 30 patients with Vancouver B1 fractures managed non-operatively were identified. Patients were matched with 60 patients with Vancouver B1 fractures who underwent surgical management, resulting in a total of 90 patients. Of the surgically managed patients, 53 out of 60 (88.3%) were treated with ORIF, while 7 out of 30 (11.7%) underwent revision THA. All seven patients who underwent revision THA showed complex fracture patterns or had a pre-fracture diagnosis of osteoporosis. A summary of cohort characteristics is provided in Table 1.

**Table 1:** Pre-Intervention characteristics

Patient demographics	Entire cohort (*n* = 90)	Nonoperative (*n* = 30)	Operative (*n* = 60)	*P* value
**Age** (SD)	84.2 (7.53)	85.0 (9.20)	83.8 (6.63)	0.53
**ASA** (SD)	3.0 (0.78)	3.3 (0.72)	2.9 (0.81)	0.83
**Gender** (Female %)	57.0 (63.3)	20.0 (66.7)	37.0 (61.7)	0.82
**Original indication** (%)				
Osteoarthritis Neck of Femur Fracture	78.0 (86.7) 12.0 (13.3)	24.0 (80.0) 6.0 (20.0)	54.0 (90.0) 6.0 (10.0)	0.43 0.31
**Implant** (%)				
Cemented Stem Uncemented Stem	41.0 (45.5) 49.0 (54.5)	14.0 (46.7) 16.0 (53.3)	27.0 (45.0) 33.0 (55.0)	0.94 0.32
**Residence on Admission** (%)				
Independent Residence	69.0 (76.7)	18.0 (60.0)	51.0 (85.0)	0.02
Nursing Home	21.0 (23.3)	12.0 (40.0)	9.0 (15.0)	0.14
**Comorbidities** (%)				
Dementia Diabetes CKD COPD IHD Prior NOF GORD AZ PD	24.0 (26.7) 27.0 (30.0) 7.0 (7.8) 9.0 (10.0) 23.0 (25.6) 8.0 (8.9) 27.0 (30.0) 3.0 (3.3) 2.0 (2.2)	12.0 (40.0) 8.0 (26.7) 5.0 (16.7) 3.0 (10.0) 10.0 (33.3) 4.0 (13.3) 16.0 (53.3) 3.0 (10.0) 2.0 (6.7)	12.0 (20.0) 19.0 (31.7) 2.0 (3.3) 6.0 (10.0) 13.0 (21.7) 4.0 (6.7) 11.0 (18.3) 0.0 (0.0) 0.0 (0.0)	0.19 0.87 0.19 1.00 0.91 0.81 0.08 0.21 0.47
**CCI** (average; SD)	6.3 (1.7)	6.9 (1.4)	6.0 (1.9)	0.04

Chronic Obstructive Pulmonary Disease (COPD); Ischemic Heart Disease (IHD); Chronic Kidney Disease (CKD); Neck of Femur Fracture (NOF); Gastroesophageal Reflux Disease (GORD); Alzheimer’s Disease (AZ); Parkinsons Disease (PD); Cemented Hemiarthroplasty (Cemented Hemi); Total Hip Arthroplasty (THA); Probability Value (p value); Charlson Comorbidity Index (CCI)

There was a statistically significant difference in CCI between the two cohorts (6.9 vs. 6.0; *P* = 0.04) showing a higher predicted 5-year mortality rate for the nonoperative group. The non-operative group had a slightly higher average age (85.0 vs. 83.8; *P* = 0.53) and a higher number of complex comorbidities. In terms of residence on admission, there was a statistically significant difference between cohorts, with a greater proportion of the operative group (85.0%) residing independently compared to the nonoperative group (60.0%; *P* = 0.02).

Table 2 summarizes the post-intervention characteristics of the two cohorts. The follow-up period differed significantly between the nonoperative and operative cohorts, with a mean of 9.9 years (SD 1.4) for nonoperative patients compared to 8.4 years (SD 2.3) for operative patients (*P* = 0.008). For residence on discharge, the operative cohort had a higher percentage of patients returning to independent living (46.7%; 6.7%; *P* = 0.001). The length of stay was similar between both cohorts, with an average of 16.6 days (SD 13.8) for the nonoperative group and 18.1 days (SD 12.8) for the operative group (*P* = 0.82). One patient initially managed nonoperatively sustained a refracture, subsequently classified as a Vancouver B2. Two months later, they underwent surgical intervention, requiring a long uncemented revision stem with cable fixation.

**Table 2:** Post-intervention characteristics

Patient demographics	Entire cohort (*n* = 90)	Nonoperative (*n* = 30)	Operative (*n* = 60)	*P* value
Length of Stay* (mean; SD)	17.6 ± 13.1	16.6 ± 13.8	18.1 ± 12.8	0.82
Follow-up** (mean; SD)	8.8 ± 2.0	9.9 ± 1.4	8.4 ± 2.3	0.008
**Weight Bearing Status ** **(%)**				
Weight Bearing as Tolerated: Non-Weight Bearing: Touch Weight Bearing: Partial Weight Bearing:	26.0 (28.9) 29.0 (32.2) 17.0 (18.9) 18.0 (20.0)	13.0 (43.0) 11.0 (36.7) 1.0 (3.3) 5.0 (16.7)	13.0 (21.7) 18.0 (30.0) 16.0 (26.6) 13.0 (21.7)	0.27 0.42 0.74 1.00
**Residence on Discharge** **(%)**				
Independent Residence Nursing Home Deceased Rehabilitation Respite	34.0 (37.8) 18.0 (20.0) 1.0 (1.1) 30.0 (33.4) 7.0 (7.7)	7.0 (23.3) 14.0 (46.7) 1.0 (3.3) 8.0 (26.7) 0.0 (0.0)	27.0 (45.0) 4.0 (6.7) 0.0 (0.0) 22.0 (36.7) 7.0 (11.6)	0.19 0.001 1.00 0.65 0.32

Weight Bearing as Tolerated (WBAT); Non-Weight Bearing (NWB); Touch Weight Bearing (TWB); Partial Weight Bearing (PWB); Standard Deviation (SD); * Length of Stay in days; ** Follow-up in years

### Mortality outcomes

There were no significant differences in mortality rates between the nonoperative and operative groups at 30 days (3.3% vs. 1.7%; *P* = 1.00) or 1 year (20.0% vs. 3.3%; *P* = 0.09). At 5 years, mortality was comparable between non-operative and operative cohorts (33.3% vs. 30.0%; *P* = 0.78). Comparative mortality rates are provided in Table 3.

**Table 3:** Mortality rates for nonoperative vs. operative cohorts

Mortality rates	Entire cohort (*n* = 90)	Nonoperative (*n* = 30)	Operative (*n* = 60)	*P* value
30 Day Mortality (%)	2.0 (2.2)	1.0 (3.3)	1.0 (1.7)	1.00
1 Year Mortality (%)	8.0 (8.8)	6.0 (20.0)	2.0 (3.3)	0.09
5 Year Mortality (%)	28.0 (31.1)	10.0 (33.3)	18.0 (30.0)	0.78

A subgroup analysis was performed to examine mortality rates based on weight-bearing status of the nonoperatively managed patients. There was no significant difference in mortality rates between WBAT and non-WBAT groups at 30 days (7.7%; 0.0%; *P* = 0.40), 1 year (15.4%; 17.6%; *P* = 0.84) and 5 years (30.8%; 35.3%; *P* = 0.78) as outlined in Table 4.

**Table 4:** Nonoperative mortality rates between WBAT and non-WBAT

Mortality outcome	WBAT (*N* = 13)	Non-WBAT (*N* = 17)	*P* value
30 Day Mortality (%)	1.0 (7.7)	0.0 (0.0)	0.40
1 Year Mortality (%)	2.0 (15.4)	3.0 (17.6)	0.84
5 Year Mortality (%)	4.0 (30.8)	6.0 (35.3)	0.78

### Power analysis

The achieved power for detecting the observed difference in mortality between the nonoperative and operative cohorts at 30 day, 1 year and 5 year follow-up was 9.2%, 71.0% and 4.8% respectively.

## Discussion

In our multicenter retrospective review of Vancouver B1 periprosthetic PPFs, we found that non-operative management resulted in comparable clinical outcomes and 30-day, 1- and 5-year mortality rates to operative treatment. Overall, postoperative weight-bearing status had minimal impact on patient mortality in the non-operative group. These findings suggest that non-operative management may offer potential benefits for select patient populations. However, further research is necessary to identify specific patient and fracture characteristics that may lead to improved outcomes for patients with PPFs managed nonoperatively [3]. 

Despite a paucity of literature, certain patient factors consistently support the viability of non-operative management, particularly a patient’s overall surgical fitness [8, 9]. In our cohort, the non-operative group exhibited a higher average predicted 5-year mortality rate, as assessed by the Charlson Comorbidity Index (CCI), compared to the operative group. Furthermore, non-operative patients were more frequently discharged to private nursing homes. At 1-year follow-up, the overall mortality rates for the nonoperative group showed rates six times higher than those of operatively managed patients (20.0% vs. 3.3%; *P* = 0.09). Given the higher CCI, increased patient age, and the greater proportion of individuals residing in nursing homes prior to the periprosthetic fracture, it may be posited that the elevated mortality at 1 year is less a reflection of the management modality employed and more a function of the patients’ underlying health status prior to the fracture event. While one might anticipate poorer long-term outcomes for the nonoperative cohort, the 5-year mortality rate, although not statistically significant, was comparatively lower in this group. These findings highlight the need to better understand rehabilitation strategies for patients once discharged into the community. The variation in discharge destinations (nursing homes, private rehabilitation) contributes to a significant degree of heterogeneity in the rehabilitation services provided, resulting in varying mortality and morbidity outcomes.

The demographic variables included in our study were selected based on their established association with poor patient outcomes following hip arthroplasty [8–10]. Factors such as advanced age, residence on admission, and comorbidities have been repeatedly shown to influence both perioperative risk and long-term survival [8–10]. Gender was a matched as a variable due to the increased fracture rates observed in post-menopausal women [12]. Matching patients by gender was crucial to improve the accuracy of our study as post-menopausal women have higher fracture incidence due to reduced bone density and hormonal changes [12]. 

While Body Mass Index (BMI) has been noted in literature as a contributor to postoperative outcomes, it was not included in our study due to data limitations. BMI was excluded as preoperative height and weight were not consistently recorded for a sufficient number of patients. A study by Park et al. (2024) identified a strong correlation between low BMI and an increased risk of hip fractures [13]. Furthermore, there was a significant correlation between the CCI, frailty, and low BMI, suggesting that more complex patients are more likely to have a higher baseline risk of fractures [13]. This highlights the compounded impact of frailty and comorbidities on fracture susceptibility, emphasizing the importance of considering these factors when assessing patient demographics in future studies to better understand their effects on outcomes.

Our study found no significant difference in mortality rates between patients permitted to weight-bear and those subjected to weight-bearing restrictions, challenging the conventional belief that early restrictions are essential for fracture healing [14, 15]. Emerging research that supports a more permissive approach, showing that permissive weight-bearing in trauma patients and immediate weight-bearing in periprosthetic fractures did not compromise outcomes, suggesting that a more liberal strategy may be safe in selected cases [14]. Growing evidence suggests that weight-bearing restrictions may not always be necessary, and in carefully selected cases, WBAT could expedite recovery, reduce immobilization-related complications, and improve patient satisfaction [9, 14–16]. In our cohort, weight-bearing restrictions stemmed from concerns about falls risk associated with complex comorbidities, including dementia, Parkinson’s disease, diabetic complications and frailty scores. Given the majority of fractures in our cohort were stable and non-displaced, there appeared to be an emphasis on early mobilization without weight-bearing restrictions for those unrestrained by complex comorbidities. To optimize outcomes, it is essential to risk stratify the benefits and risks within patient groups prior to determining specific weight-bearing protocols.

However, our study has several limitations. As a retrospective cohort study with a small sample size, it is susceptible to selection bias and confounding factors [17]. Non-operatively managed patients were older, had a greater burden of comorbidities and were more frequently discharged to nursing facilities, suggesting that treatment decisions were potentially influenced by patient factors, rather than fracture characteristics alone. Additionally, the low statistical power limits our ability to detect significant differences in mortality outcomes between nonoperative and operative management of Vancouver B1 periprosthetic fractures. The relatively small sample sizes and marginal mortality differences reduce the likelihood of drawing definitive conclusions. To address these limitations, future research should adopt a multicenter, prospective cohort design with a larger and more diverse sample size. Systematic data collection on treatment protocols, patient demographics, and long-term outcomes will help control variables and minimize selection bias, facilitating the development of standardized processes for nonoperative management. Based on our power calculations for five-year mortality, approximately 2,300 nonoperative and 5,000 operative patients would be needed to achieve 80% power, ensuring sufficient sensitivity to detect meaningful differences. Larger studies with extended follow-up periods are necessary to provide robust evidence and guide clinical practice.

Accurately distinguishing between Vancouver B1 and B2 periprosthetic fractures posed a challenge in our study. Although we applied standardized clinical and radiographic criteria for preoperative fracture classification, intraoperative assessment remains the gold standard for determining prosthetic loosening [4]. Notably, seven of the sixty patients who underwent operative management were treated with revision THA, suggesting that intraoperative findings may have revealed previously undetected implant instability, thereby potentially reclassifying these fractures as Vancouver B2. However, there were no explicit references to prosthetic loosening in the postoperative reports for these cases. This raises the possibility that the decision to revise the femoral component was influenced not only by intraoperative findings but also by surgeon preference and broader clinical considerations, including patient comorbidities and functional status. These findings highlight the inherent limitations in pre-operative fracture classification and emphasize the need for clear documentation and justification of intraoperative decision-making. Ensuring accurate reclassification of fractures when intraoperative findings contradict initial assessments is essential for improving the reliability of research outcomes and guiding evidence-based treatment strategies.

Our study also had limitations accessing patient follow-up images. We could only access images available through patients’ electronic medical records (EMR) and public hospital imaging platforms in our district. Despite efforts to obtain all images from private providers, we successfully retrieved only eight out of 30 (26.7%). Previous studies have provided insights into fracture patterns suitable for non-operative management [8, 9, 18]. A study by Efird et al. (2023) identified a potential correlation between the distance of the fracture from the tip of the prosthesis stem, finding that all nonoperatively managed patients without fracture displacement had fractures averaging roughly ten centimeters proximal to the stem [9]. In our cohort, 6 out of the 8 follow-up images showed non-displaced and united fracture patterns at an average follow-up of 7.2 months. Interestingly, these patterns were consistent with those outlined in previous studies [8, 9]. Overall, the limitations we encountered in collecting follow-up radiographs highlight the need for a secure, centralized database patient medical imaging. This would not only expedite care coordination between multi-disciplinary teams but also facilitate access for future research, enabling more definitive conclusions on hypothesized outcomes and advancing our understanding of fracture management strategies.

Finally, a significant limitation of our study is the absence of patient-reported outcome measures (PROMs) to evaluate the overall impact of treatment on patient morbidity between the nonoperative and operative groups managing PPFs. While we collected mortality data, this metric alone does not encompass the broader implications of fracture management, such as functional status, pain levels, and quality of life. Research indicates that PROMs are essential for understanding the patient’s experience and treatment effectiveness, particularly in orthopedic contexts where functional recovery is paramount [19, 20]. The lack of PROMs in our analysis limits our ability to draw comprehensive conclusions about the true burden of morbidity associated with different treatment strategies for PPFs. Future studies should prioritize the inclusion of PROMs to capture these critical dimensions of patient health and recovery, leading to more patient-centered approaches in clinical decision-making [19, 20]. h.

## Conclusion

In conclusion, our study demonstrates that a subset of patients with high frailty and significant comorbidities may be safely managed non-operatively for stable Vancouver Type B1 periprosthetic femur fractures. These findings suggest that non-operative treatment may be a viable option in carefully selected patients; however, the ideal patient population and optimal rehabilitation strategy remain unclear. Further research is necessary to refine selection criteria and establish evidence-based guidelines to improve outcomes for patients undergoing non-operative management of periprosthetic femur fractures.

## Data Availability

The datasets generated and/or analysed during the current study are not publicly available due to the terms of our ethics approval but are available from the corresponding author on reasonable request.

## Abbreviations

PPF: Periprosthetic Femoral Fractures
WBAT: Weight Bearing as Tolerated
THA: Total Hip Arthroplasty
NOF: Neck of Femur Fracture
OA: Osteoarthritis
ASA: American Society of Anesthesiologists
SD: Standard Deviation
COPD: Chronic Obstructive Pulmonary Disease
CKD: Chronic Kidney Disease
IHD: ischemic heart disease
NWB: Non-Weight Bearing
TWB: Touch Weight Bearing
PWB: Partial Weight Bearing
SD: Standard Deviation
EMR: Electronic Medical Records
PROMs: Patient-Reported Outcome Measures
CCI: Charlson Comorbidity Index
